# The Magnitude of Physical and Sensory Impairments in Post-traumatic and Non-traumatic Cervicogenic Headaches: A Comparative Study

**DOI:** 10.7759/cureus.47146

**Published:** 2023-10-16

**Authors:** Mosab Amoudi, Qais Nairat

**Affiliations:** 1 Faculty of Allied Medical Sciences, Arab American University, Jenin, PSE; 2 Faculty of Educational Sciences and Teachers’ Training, An-Najah National University, Nablus, PSE

**Keywords:** post-trauma, physical impairment, movement, migraine, cervicogenic headache

## Abstract

Background

Cervicogenic headaches can significantly deteriorate the quality of life of patients and decrease their productivity. Few studies have been conducted to assess the magnitude of physical impairments among patients with non-traumatic and post-traumatic cervicogenic headaches. Therefore, this study was conducted to examine and compare the magnitude of cervical physical impairments among patients with post-traumatic cervicogenic headaches in contrast to patients with non-traumatic cervicogenic headaches, migraine, and a group of sex- and age-matched controls who did not have headaches.

Methodology

This was a comparative study. A total of 104 patients and 30 sex- and age-matched controls were included. The patients were stratified into three groups: post-traumatic cervicogenic headaches (n = 42), non-traumatic cervicogenic headaches (n = 30), and migraines (n = 32). The cervical active range of motion and proprioception were assessed using a goniometer, the craniovertebral angle was measured while the heads of the subjects rested in a comfortable position, the strength of the cervical flexors and extensors was assessed using a hand-held dynamometer, and endurance of the short cervical flexors was assessed while the subjects were in a supine position with no pillow. Skin roll tests were performed in the trapezius and mandibular areas, and pain was measured using the McGill Pain Questionnaire-Short Form.

Results

Patients in the post-traumatic cervicogenic headache group reported a significantly higher number of headache days (p < 0.001) compared to the controls, patients with migraines, and those with non-traumatic cervicogenic headaches. Patients with post-traumatic cervicogenic headaches showed a significant reduction in total rotation, flexion and extension, lateral bending, and craniovertebral angle compared to the controls, patients with migraines, and those with non-traumatic cervicogenic headaches. On the other hand, the controls endured significantly longer and had stronger neck flexors and extensors compared to the patients in the migraine, non-traumatic, and post-traumatic cervicogenic headache groups, respectively. Patients with post-traumatic cervicogenic headaches significantly reported higher affective and total pain compared to the controls and patients in the migraine group.

Conclusions

Patients with post-traumatic cervicogenic headaches had significant deficits in the cervical active range of motion in the different planes, endurance, and strength of cervical flexors and extensors compared to the controls and patients with migraine and non-traumatic cervicogenic headaches. Similarly, patients with post-traumatic cervicogenic headaches reported higher affective pain compared to the controls and patients with migraines. The results of this study indicated that patients with post-traumatic cervicogenic headaches have significantly higher physical impairments compared to patients with non-traumatic cervicogenic headaches. These differences warrant caution when combining data from patients with non-traumatic and post-traumatic cervicogenic headaches.

## Introduction

Cervicogenic headaches are symptomatic unilateral chronic headaches that usually arise from the upper cervical spine [1]. They are defined as headaches caused by the cervical spine, the bony components of the cervical spine, disc, and/or soft tissues [2]. Cervicogenic headaches are often but not invariably coupled with neck pain. It is widely thought that cervicogenic headaches are secondary to primary cervical musculoskeletal disorders [2]. These disorders may involve lesions in the cervical intervertebral disks, uncovertebral joints, nerves and nerve roots, muscles, or ligaments. Cervicogenic headaches can be chronic or present as episodes that are accompanied by neck stiffness and pain. Among adults, previous studies have reported that the one-year prevalence rates of cervicogenic headaches ranged from 0.2% to 2.2% [3].

It has been argued that the late changes in lifestyle have contributed to the incidence of cervicogenic headaches [4]. It is widely thought that sitting in poor postures, including slouched posture, forward head posture, prolonged neck flexion, and/or poor ergonomics, can cause muscular imbalance and joint stiffness that would later lead to increased pain and tension in the cervical muscles [2]. Similarly, the risks of developing cervicogenic headaches are thought to be increased by repetitive neck movements, including those performed while performing computer, bureau, or manual work-related tasks [2,4]. Many previous studies have reported a high prevalence of neck, shoulder, and upper back pain among office-based employees. In a study in Jordan, about one-third of office-based employees reported neck, shoulder, and lower back pain [4]. Moreover, cervicogenic headaches could be attributed to sustaining a traumatic event such as a motor vehicle accident [2]. According to the 2020 statistics of the US National Highway Traffic Safety Administration, 1,593,390 motor vehicle accidents resulted in injuries [5]. Previous studies have reported that injuries to the neck or paravertebral soft tissues are associated with cervicogenic headaches [2]. It is well-established that cervicogenic headaches can significantly deteriorate the quality of life of patients and decrease their productivity [4].

Symptoms of cervicogenic headaches overlap with the symptoms of other types of headaches. Because cervicogenic headaches are often unilateral, they can be mistaken for migraines. On the other hand, cervicogenic headaches are often described by patients as dull, aching, or throbbing pain, and can be mistaken for tension-type headaches [2]. It is noteworthy to mention that cervicogenic headaches arise from the neck and radiate to the head. Moreover, cervicogenic headaches could be triggered or even worsened by some neck positions or movements. Therefore, neck pain, stiffness, and restricted cervical range of movement are common in cervicogenic headaches [2]. On the other hand, migraines can be severe, throbbing, or pulsating headaches. Nausea, photophobia, and phonophobia have also been reported by patients with migraines. On the other hand, tension-type headaches are less severe than migraines and often described as dull, aching, or pressure-like pain. This makes the clinical diagnosis and characterization of this type of headache difficult. Relying on the patients to describe their symptoms is not a reliable approach for accurate diagnosis of cervicogenic headaches [2,6]. So far, few objective tests have been proposed to help diagnose cervicogenic headaches. These objective tests include diagnostic blocks. It is noteworthy to mention that these diagnostic blocks require specialized facilities, should be performed when the patients are experiencing pain, and are invasive [2,6]. Alternatively, detecting cervical physical impairments has been suggested to help diagnose cervicogenic headaches.

It is widely thought that cervicogenic headaches can be treated by rehabilitating the musculoskeletal impairments that cause the pain [6]. A recent systematic review reported that manual therapeutic techniques were effective in the management of cervicogenic headaches [7]. Manual therapeutic techniques included muscle techniques, Mulligan’s Sustained Natural Apophyseal Glides, spinal manipulative therapy, and translatory vertebral mobilization. Of the different techniques, compressing the sternocleidomastoid and Jones technique on the trapezius achieved immediate alleviation of cervicogenic headaches [7]. Previous studies have reported that patients suffering from cervicogenic headaches had reduced endurance and strength of the short neck flexors and extensors and displayed different head postures when compared with others who did not have cervicogenic headaches [2,6]. Similarly, restricted active cervical range of motion was also reported among patients who had cervicogenic headaches [6]. Moreover, deficits in the proprioception of the neck were also reported among patients who suffered motor vehicle accidents and those who had chronic neck pain [6,8]. Few studies have been conducted to examine proprioception of the neck in patients with cervicogenic headaches [6]. Pain upon palpation has also been reported among patients with different headache types including those who had cervicogenic and migraine headaches [9]. Similarly, different patterns of pain including sensory and affective pain have been previously reported among patients with different types of headaches [2]. However, few studies have been conducted to assess the different patterns of pain among patients with cervicogenic headaches [6].

So far, few studies have been conducted to assess the magnitude of physical impairments among patients who have cervicogenic headaches that could be related to a traumatic event (non-traumatic cervicogenic headaches) and those who have cervicogenic headaches that could not be related to a traumatic event (post-traumatic cervicogenic headaches), notably, in developing countries and resource-limited settings. Therefore, this study was conducted to examine and compare the magnitude of cervical physical impairments among patients with post-traumatic cervicogenic headaches in contrast to patients with non-traumatic cervicogenic headaches, migraine, and a group of sex- and age-matched controls who did not have headaches.

## Materials and methods

Study design

This was a comparative study that compared the magnitude of physical impairments in terms of restricted cervical active range of motion, cervical proprioception, craniovertebral angle, weakened strength of the cervical flexors and extensors, endurance of the short cervical flexors, and increased pain among patients with post-traumatic cervicogenic headaches in contrast to patients with non-traumatic cervicogenic headaches, migraines, and a group of sex- and age-matched controls who did not have headaches [6]. This study was conducted in habilitation centers in a resource-limited setting in a developing country. Resource-limited settings are inherently deficient with professionals, infrastructure, and equipment needed to provide quality rehabilitation services.

Study subjects

In this study, a total of 104 patients and 30 matched controls were included. Of the patients, 32 were already diagnosed with migraine by a licensed neurologist as per the International Headache Society (IHS) criteria [2]. Another 72 patients were already diagnosed with cervicogenic headache by a licensed neurologist as per the IHS criteria [2]. The criteria used in the diagnosis of cervicogenic headaches included (1) neck pain that was associated with the onset or preceded by headaches, (2) the episodes of headache were aggravated and/or precipitated by the sustained posture of the neck, (3) the presence of abnormal pain upon palpation of the neck, and (4) restricted cervical active range of motion [2,6,10]. Of the patients who had cervicogenic headaches, the onset of headaches could not be related to any trauma in 30 patients. On the other hand, 42 patients reported a traumatic event (a motor vehicle accident) at the onset of their headache. For all patients, the trauma did not result in a concussion or amnesia as determined by a licensed neurologist. In this study, all clinical assessments and diagnoses were made by neurologists who were licensed to practice. The neurologists assessed the patients recruited for this study against the inclusion and exclusion criteria. The patients were included in this study if they were ≥18 years old, had a diagnosis of migraine or cervicogenic headaches (regardless of whether their headaches could be related to a traumatic event or not), and had symptoms for more than six months. Patients were excluded if they were diagnosed with a systemic or central nervous disease. The study subjects in the control and migraine groups were excluded if they reported a history of cervical pain that affected the course of their normal daily activity in the last five years. All patients were recruited from rehabilitation centers.

The controls were invited, screened, and recruited from the local society to sex- and age-match the patients in the migraine and cervicogenic headache groups.

The study was conducted in compliance with local and international ethical principles followed in scientific research including those in the Declaration of Helsinki. The study was approved by the Institutional Review Board of An-Najah National University (approval number: IRB-14-Sep-21), and written informed consent was provided by all subjects.

Assessment of the subjects

In this study, the following assessments were performed: cervical active range of motion, cervical proprioception, craniovertebral angle (posture), strength of the cervical flexors and extensors, endurance of the short cervical flexors, and pain.

Cervical active range of motion

The cervical active range of motion was measured using a goniometer (CROM goniometer, Performance Attainment Associates, Roseville, MN). The reliability of this technique was acceptable, as indicated by an intraclass correlation coefficient of 0.76 to 0.98 [11]. During measurements, the subjects were maintained sitting while the feet were maintained flat on the floor. The thoracic spine was in contact with the backrest of the chair while the lumbar spine was positioned in neutral. Rotation, flexion, extension, and lateral bending were bilaterally measured and repeated twice. For the frontal and transverse planes, movements to both sides were summed. Similarly, for the sagittal plane, flexion and extension were combined, summed, and averaged, as previously described [6,12].

Cervical proprioception

Cervical proprioception was measured using a goniometer. Similar to the cervical active range of motion, the reliability of the goniometer in assessing cervical proprioception was previously reported as acceptable [11]. The goniometer was placed on the heads of the subjects keeping their eyes closed. The examiner then positioned the heads of the subjects at a 30° right rotation and then the heads were passively turned to 0° [13]. This position was reproduced three times and a measurement was made each time. The same procedure was iterated in five other positions, namely, left rotation 30°, left rotation 50°, left lateral bending 20°, right rotation 50°, and right lateral bending 20° [6].

Posture

Posture was measured using the craniovertebral angle. This angle was formed by a horizontal line that passed through the spinous process of C7 and a line that passed through the tragus of the ear and again the spinous process of C7 [6,14]. A digital high-quality photograph was taken laterally while the subjects were sitting in this position. Before the photographs were taken, the subjects were asked to perform flexion and extension movements of large amplitude. The subjects were then asked to reduce the amplitudes of the movements to the point that the heads rested in comfortable positions for the subjects [15]. A photographic edition software (CorelDRAW Graphics Suite, Alludo, Ottawa, Ontario, Canada) was used to measure the angle.

Strength of the cervical flexors and extensors

The strength of the cervical flexors and extensors was measured using a hand-held dynamometer (microFET®2, Hoggan Scientific, Salt Lake City, UT) [16]. The reliability of this technique was shown to be good, as indicated by an intraclass correlation coefficient of more than 0.74 [17]. The subjects were positioned in the supine position and the chin was retracted. The dynamometer was placed on the center of the forehead keeping the head slightly off the bed. The subjects were requested to push against the dynamometer using maximal force while the examiner was holding the dynamometer (isometric hold). To measure the strength of the cervical extensors, the subjects were asked to lie down in a prone position. The dynamometer was placed in the back of the head. The subjects were asked to perform contractions against the dynamometer that was held by the assessor. The contractions lasted three to five seconds. The measurements were repeated twice with a 60-second rest between the rounds.

Endurance of the short cervical flexors

Endurance of the short cervical flexors was examined while the subjects were in a supine position with no pillow [18]. The heads of the subjects were passively positioned 2 cm away from the plinth while the chins were retracted. The subjects were asked to hold their heads still for as long as they could. The length of time, until the chins began to thrust, was measured using a stopwatch.

Pain

The skin in the trapezius and mandibular areas was rolled in the skin roll test. The trapezius area was halfway between the C6 process and the acromion and the mandibular area was at the angle of the jaw. The subjects were asked to indicate the pain caused by the skin roll using a visual analog scale (VAS) of 100 mm. The skin was rolled once on each side of the body.

The subjects were asked to complete the McGill Pain Questionnaire-Short Form (MGPQ-SF) [19,20]. The questionnaire measured a present pain intensity score (0-5) in addition to the affective and sensory components of the pain. Total pain combined both affective and sensory components of pain to provide insights into the overall pain experience of the subjects. In this study, the subjects were asked to rate the most frequent headaches they usually suffered.

Statistical analysis

The data were entered into GraphPad Prism v.7.0. The data were tested for normal distribution using the Shapiro-Wilk test. Statistically significant differences between the groups were tested using the one-way analysis of variance. To control for type 1 and type 2 errors, a significance level of 0.05 and Tukey’s post-hoc tests were used. The sample size used in this study was calculated at a power of >80% and was larger than those used in previous studies [6,8,14,21].

## Results

Characteristics of the subjects

There were no statistically significant differences in the age and sex distribution of the patients in the different groups. The patients in the post-traumatic headache group had experienced their trauma for a mean of 7.4 ± 6.7 years, and the patients in the non-traumatic headache group had their symptoms for a mean of 13.9 ± 11.8 years. The onset of cervicogenic headaches in the post-traumatic headache group ranged from 0 to 11 months. Patients in the post-traumatic cervicogenic headache group reported a significantly higher number of headache days (p < 0.001) compared to the patients in the other groups. Similarly, the patients who had migraines reported significantly lower number of headache days (p < 0.001) compared to the patients in the cervicogenic headache groups. The characteristics of the study subjects are shown in Table 1.

**Table 1:** Characteristics of the subjects. CGH: cervicogenic headache; SD: standard deviation; n/a: not applicable

Group	n	Age, mean ± SD (years)	Male/female	Days of headache/month
Control	30	46.2 ± 13.4	16/14	n/a
Migraine	32	44.5 ± 12.8	14/18	6.5 ± 4.2
CGH	72	43.6 ± 11.6	35/37	21.6 ± 8.6
Non-traumatic CGH	30	45.4 ± 14.3	13/17	17.4 ± 11.2
Post-traumatic CGH	42	42.8 ± 10.7	22/20	28.3 ± 6.2

**Figure 1:**
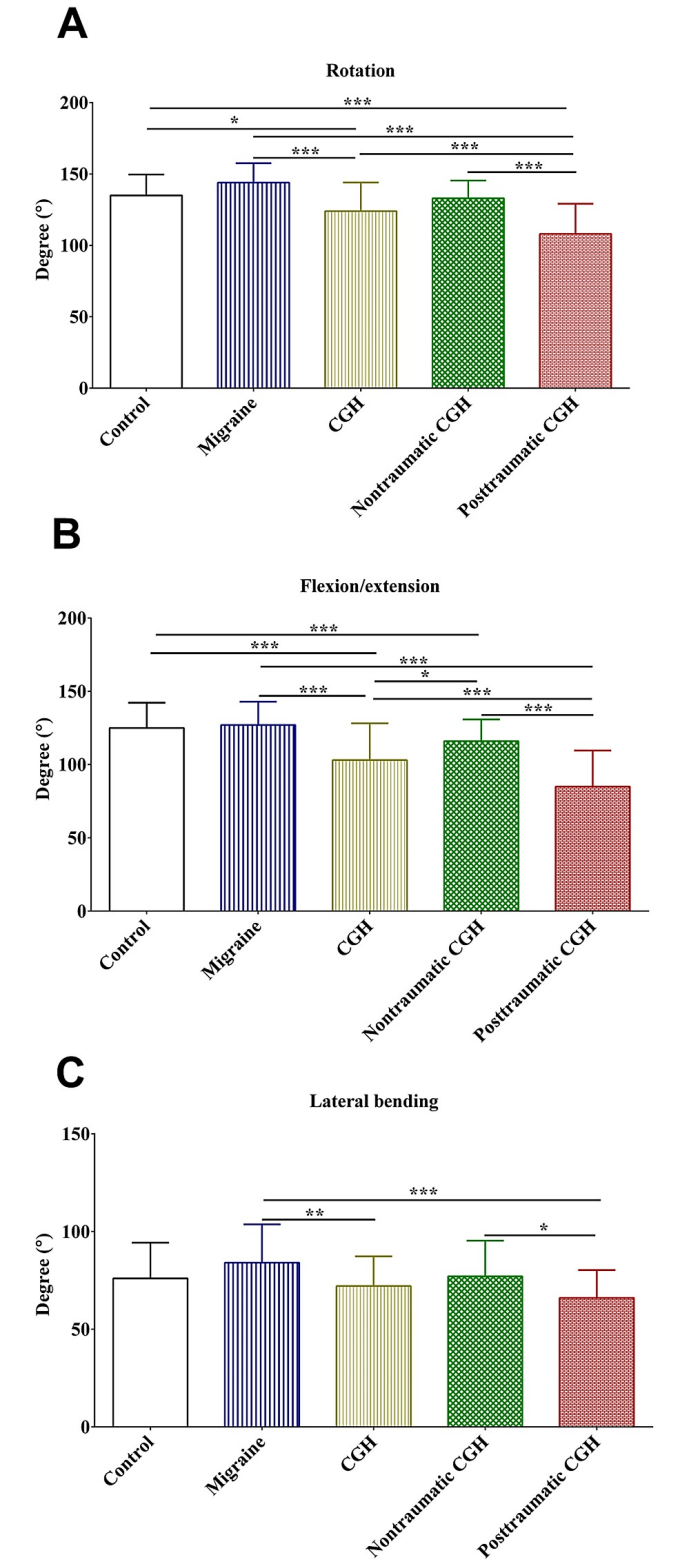
Cervical active range of motion. Total rotation (A), flexion/extension (B), and lateral bending (C). CGH: cervicogenic headache; *: p-value <0.05; **: p-value <0.01; ***: p-value <0.001

In this study, there were statistically significant differences in the active range of motion between the study patients in the different groups, as shown in Figure 1. The total rotation (movement performed in the transverse plane) was significantly reduced in patients who had cervicogenic headaches compared to the controls and those who had migraines (Figure 1, Panel A). Moreover, the patients who had post-traumatic cervicogenic headaches showed further reduction in total rotation compared to those who had non-traumatic cervicogenic headaches (Figure 1, Panel A). On the other hand, there was no statistically significant difference in total rotation between the controls and those who had migraine headaches.

The total flexion and extension (movement performed in the sagittal plane) were significantly reduced in patients who had cervicogenic headaches compared to the controls and those who had migraines (Figure 1, Panel B). Additionally, the patients who had post-traumatic cervicogenic headaches showed further reduction in total flexion and extension compared to those who had non-traumatic cervicogenic headaches (Figure 1, Panel B). Similar to total rotation, there was no statistically significant difference in total flexion and extension between the controls and those who had migraine headaches.

The total lateral bending (movement performed in the frontal plane) was significantly reduced in patients with cervicogenic headaches compared to those who had migraines (Figure 1, Panel C). In addition, there was a further significant reduction in lateral bending among patients who had post-traumatic cervicogenic headaches compared to those who had non-traumatic cervicogenic headaches (Figure 1, Panel C). Similar to the movements performed in the transverse and sagittal planes, there were no statistically significant differences in total lateral bending between the controls and those who had migraine headaches.

Proprioception

**Figure 2:**
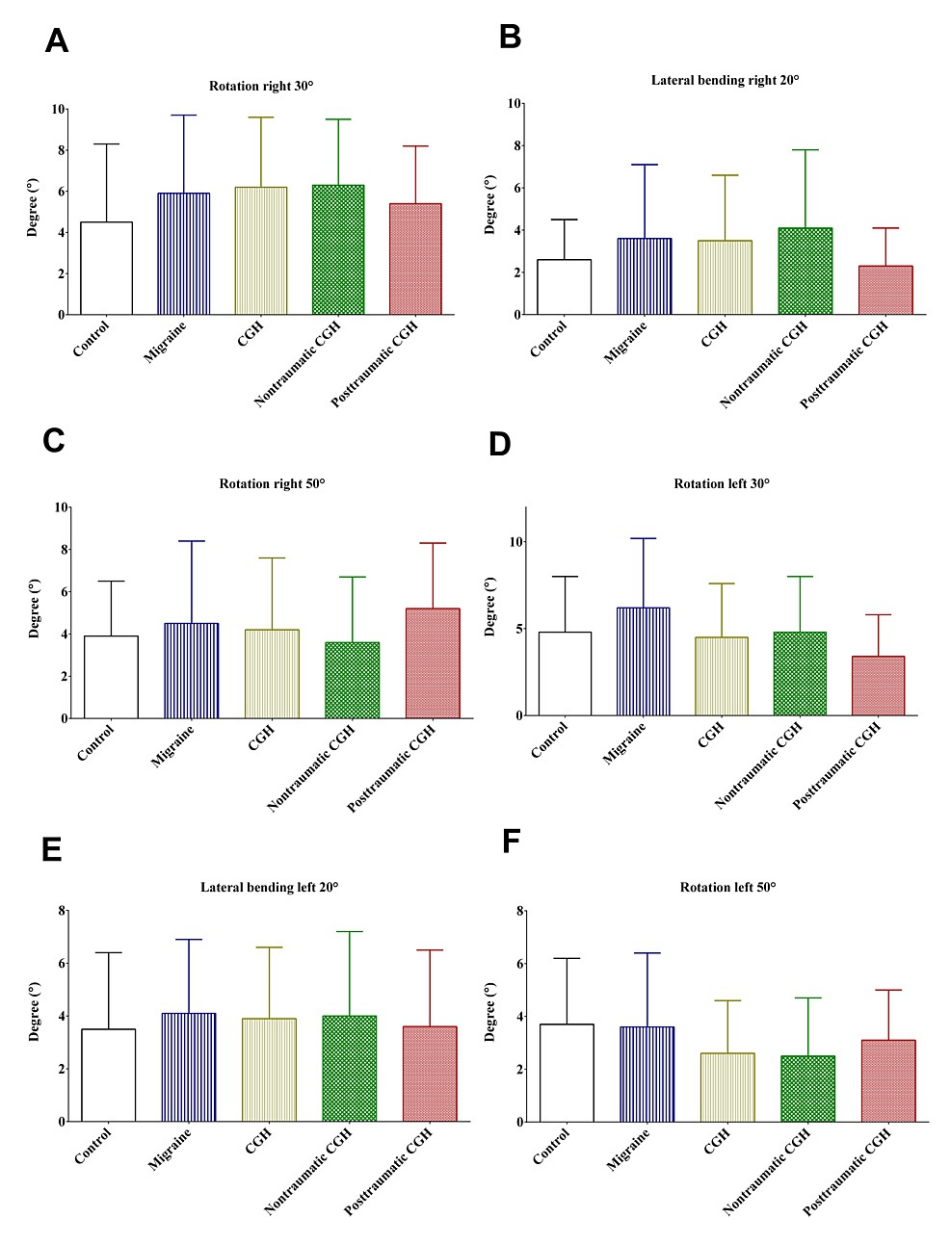
Error in repositioning (proprioception) of the head for rotation and lateral bending on right (A-C) and left (D-F) sides. CGH: cervicogenic headache; *: p-value <0.05; **: p-value <0.01; ***: p-value <0.001

In this study, there was no statistically significant difference in the right and left rotation or lateral bending in different degrees between the patients from the different groups, as shown in Figures 2A-2F.

Posture, endurance, and strength of neck flexors and extensors

**Figure 3:**
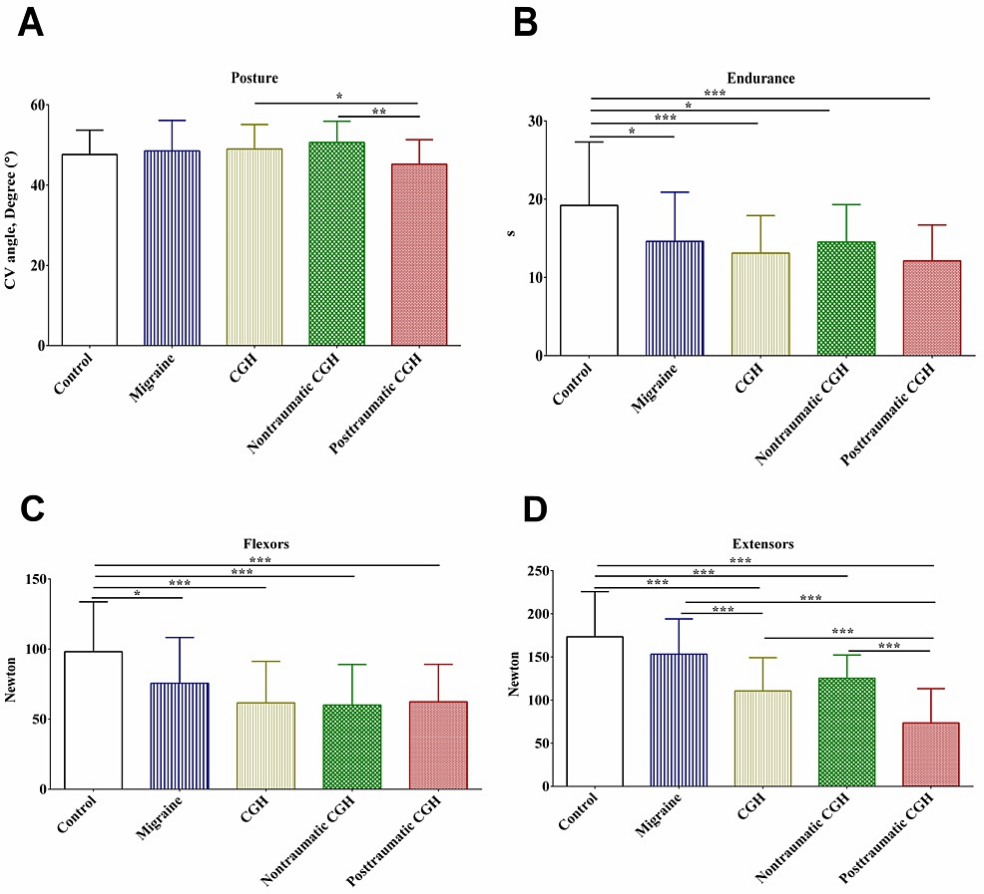
Craniovertebral angle (A), endurance of the short cervical flexors (B), and strength of the cervical flexors (C) and extensors (D). CGH: cervicogenic headache; CV: craniovertebral angle; *: p-value <0.05; **: p-value <0.01; ***: p-value <0.001

In this study, there were significant differences in the craniovertebral angle (posture) between patients who had post-traumatic cervicogenic headaches compared to those who had non-traumatic cervicogenic headaches, as shown in Figure 3, Panel A. On the other hand, there were no statistically significant differences in the craniovertebral angle between patients who had migraines, non-traumatic cervicogenic headaches, and the controls (Figure 3, Panel A).

When endurance was compared, the controls endured significantly longer than the other patients in the migraine, non-traumatic, and post-traumatic cervicogenic headache groups, as shown in Figure 3, Panel B. Although there was a trend of reduced endurance among patients who had migraine, non-traumatic, and post-traumatic cervicogenic headaches, this trend was not statistically significant (Figure 3, Panel B).

Similar to endurance, the controls showed statistically stronger neck flexors compared to the patients in the migraine, non-traumatic, and post-traumatic cervicogenic headache groups, as shown in Figure 3, Panel C. Again, there was a trend of reduced strength of neck flexors among patients who had migraine, non-traumatic, and post-traumatic cervicogenic headaches; however, this trend was not statistically significant (Figure 3, Panel C).

In this study, the controls showed significantly stronger neck extensors compared to the patients in the migraine, non-traumatic, and post-traumatic cervicogenic headache groups, as shown in Figure 3, Panel D. There was a statistically significant trend of reduced strength of neck extensors among patients who had migraine, non-traumatic, and post-traumatic cervicogenic headaches, in which the latter exhibited the least strength of neck extensors compared to the other groups (Figure 3, Panel D).

Pain

**Figure 4:**
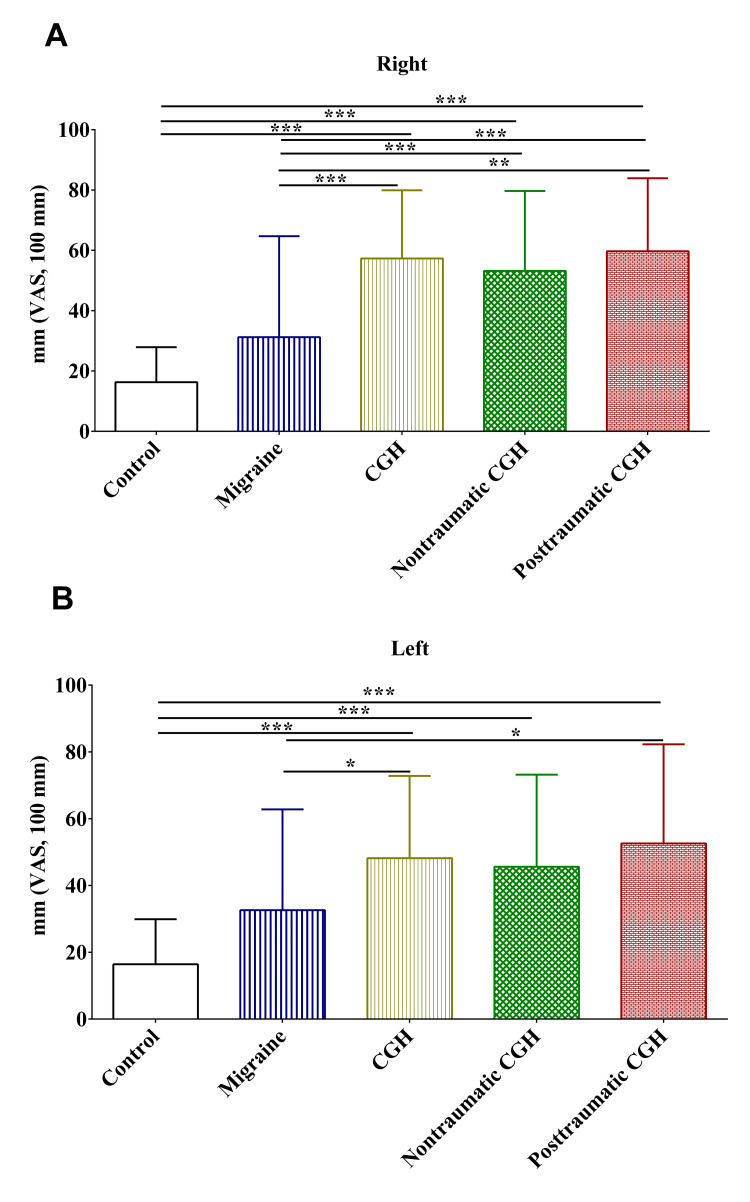
The 100 mm visual analog scale completed by the subjects after the skin roll test on the right (A) and left (B) sides of the trapezius area. CGH: cervicogenic headache; VAS: visual analog scale; *: p-value <0.05; **: p-value <0.01; ***: p-value <0.001

Using a 100 mm VAS, patients in the migraine, non-traumatic, and post-traumatic cervicogenic headache groups reported significantly higher pain in the right and left trapezius areas compared to the controls (Figures 4A, 4B). Pain in the right and left trapezius areas was not statistically different. Compared to those in the migraine group, patients in the non-traumatic, and post-traumatic cervicogenic headaches reported significantly higher pain in the right and left trapezius areas (Figures 4A, 4B).

**Figure 5:**
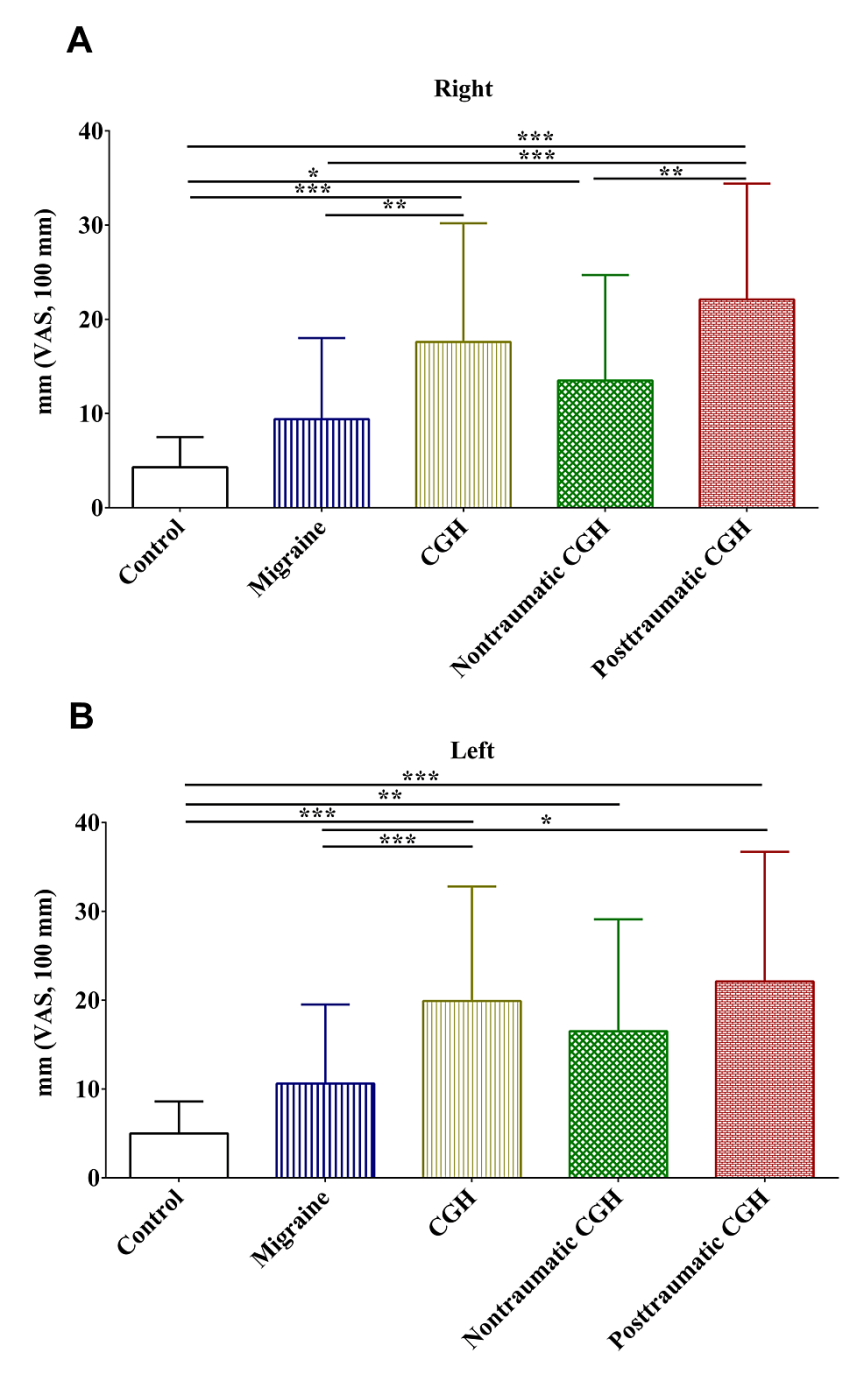
The 100 mm visual analog scale completed by the subjects after the skin roll test on the right (A) and left (B) sides of the mandibular area. CGH: cervicogenic headache; VAS: visual analog scale; *: p-value <0.05; **: p-value <0.01; ***: p-value <0.001

Similarly, patients in the migraine, non-traumatic, and post-traumatic cervicogenic headache groups reported significantly higher pain in the right and left mandibular areas compared to the controls (Figures 5A, 5B). Compared to the patients in all groups, patients in the post-traumatic cervicogenic headache group reported higher pain.

**Figure 6:**
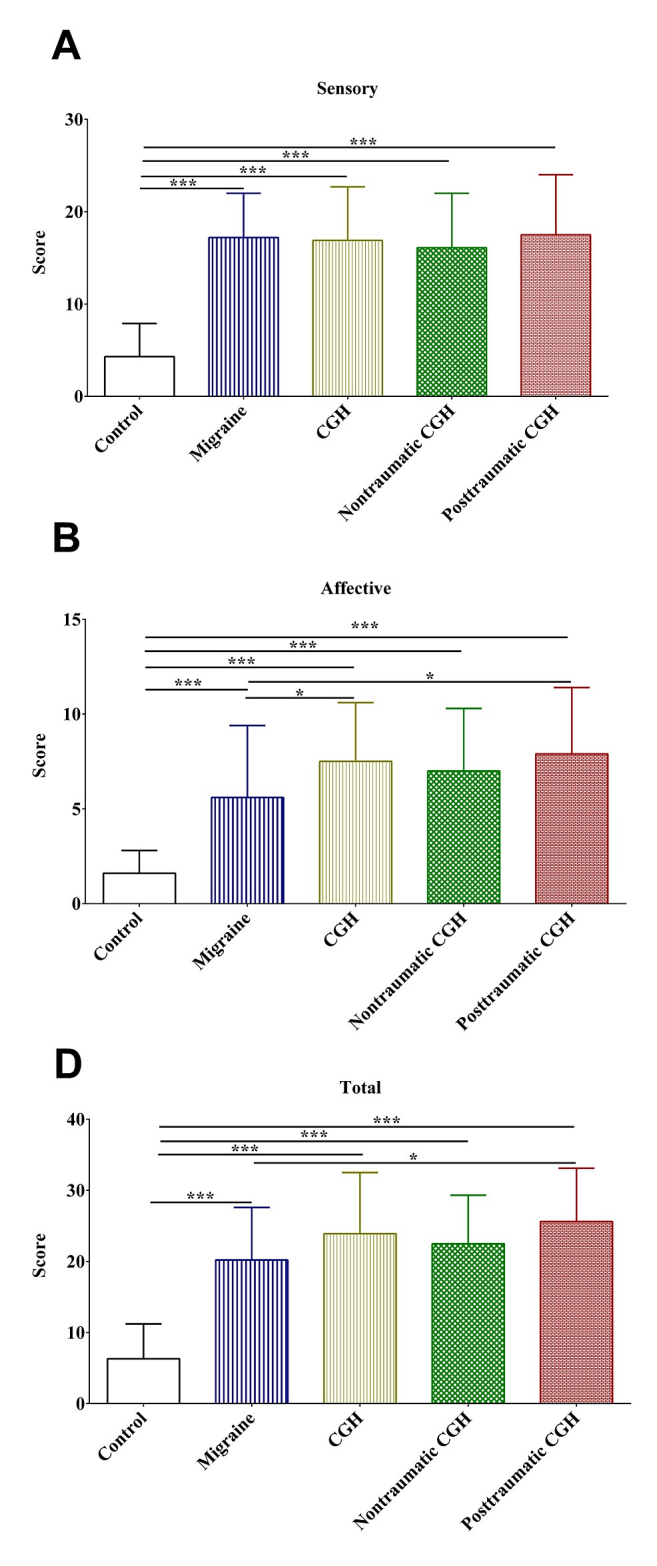
Pain ratings on the McGill Pain Questionnaire-Short Form on the sensory (A), affective (B), and total (C) components. CGH: cervicogenic headache; *: p-value <0.05; **: p-value <0.01; ***: p-value <0.001

Again, patients in the migraine, non-traumatic, and post-traumatic cervicogenic headache groups reported significantly higher sensory, affective, and total pain compared to the controls, as measured using the MGPQ-SF (Figure 6A-6C). Additionally, patients who had post-traumatic cervicogenic headaches reported higher affective and total pain compared to patients in the migraine group (Figures 6B, 6C).

## Discussion

Cervicogenic headaches are prevalent types of headache that affect 1% to 4% of headache patients [1]. Few studies have been conducted to examine the magnitude of physical impairments among patients with cervicogenic headaches, notably in developing countries and resource-limited settings. To our knowledge, this is the first study that was conducted to assess and compare the magnitude of physical impairments among patients with cervicogenic headaches that could be or could not be linked to a traumatic event in comparison to those who had migraines and a group of controls who did not have headaches recruited in a resource-limited setting. The findings of this study might help understand physical impairments in cervicogenic headaches. The findings of this study are informative for neurologists, physiotherapists, general practitioners, and other healthcare providers who care for patients with cervicogenic headaches.

The findings of this study showed that patients who had post-traumatic cervicogenic headaches had significantly reduced cervical active range of motion. This was obvious for the rotations (movements in the transverse plane) and flexion and extension (movements in the sagittal plane) when compared to the patients who had non-traumatic cervicogenic headaches, migraines, and controls. The findings reported in this study were consistent with those reported in previous studies [6,12]. Similar to previous studies [6,22], the cervical active range of motion in patients with migraine was not different from the controls. These findings indicated that reduced cervical active range of motion is a distinctive characteristic of post-traumatic cervicogenic headaches. Probably, this could be explained by the extent of the musculoskeletal impairments caused by the traumatic event to which the patients were subjected. Therefore, rehabilitating these musculoskeletal impairments that cause the pain could improve the cervical active range of motion in this distinct group of patients [6,7]. These findings might be helpful in the diagnosis of post-traumatic cervicogenic headache and in measuring the magnitude of physical impairments among the patients.

Although there was a decrease in cervical proprioception among patients who had post-traumatic cervicogenic headaches in the majority of the positions, this decrease was not statistically significant. These findings are consistent with those that used the same technique [6]. It is noteworthy to mention that cervical proprioception can decrease as a result of traumatic injuries, neurological disorders, or other diseases that affect the sensory receptors [1-3,6]. Improving proprioceptive awareness through specifically designed exercises and rehabilitations might improve proprioceptive deficits. On the other hand, the findings reported in this study were contradictory to those that reported a significant reduction in cervical proprioception among patients who had post-traumatic cervicogenic headaches [8,13,20]. These contradictory findings could be explained by the nature of trauma and the extent of post-traumatic impairments caused [23].

In this study, there was a significant reduction in the craniovertebral angle as a measure of posture in patients who had post-traumatic cervicogenic headaches compared to patients who had non-traumatic cervicogenic headaches. These findings were consistent with those reported in a previous study [24]. However, a previous study failed to detect significant differences in the craniovertebral angle among patients who had post-traumatic cervicogenic headaches compared to patients who had non-traumatic cervicogenic headaches [6]. These differences could be explained by the larger sample size used in this study and the complexity of human head posture.

The findings of this study showed significant deficits in the strength and endurance of short neck flexors and extensors. The findings reported in this study were consistent with those reported in previous studies that used similar techniques and those that used laboratory equipment [6,24,25]. Taken together, these findings indicate deficits in the strength and endurance of short neck flexors and extensors are characteristics that could be used to measure the magnitude of physical impairments among patients.

Consistent with the findings of previous studies, there was a significant increase in pain caused by skin roll tests on both sides of the trapezius mandibular areas [6,25]. Together, these findings might highlight the utility of this test in detecting physical impairments in headaches, notably in cervicogenic headaches. Probably, more studies are still needed to establish the reliability of the skin roll test and to validate it against the algometry of pressure pain. On the other hand, the findings of this study were contradictory to those reported in a previous study in which the MGPQ-SF failed to discriminate sensory and affective components of pain in patients with migraine, non-traumatic cervicogenic headaches, and post-traumatic cervicogenic headache [6]. In this study, the MGPQ-SF could discriminate pain, notably affective pain among patients in the different groups, with post-traumatic cervicogenic headache patients reporting the highest pain. These contradictory results might be explained by the larger sample size in the different groups compared in this study. The findings of this study warrant further investigation into the utility of the MGPQ-SF in assessing affective pain in post-traumatic cervicogenic headaches. Probably, the MGPQ-SF can be used for the assessment of affective pain among patients with post-traumatic cervicogenic headaches.

Strengths of the study

This study has many strong points that might be considered when interpreting the results. First, this is the first investigation of the magnitude of physical impairments among patients with cervicogenic headaches with or without traumatic events in a resource-limited country. The findings of this study might strengthen those previously reported in other settings. Second, the sample size used in this study was larger than those used in the previous studies [6]. It is well-established that findings reported from larger studies are more reliable than those reported from studies with small sample sizes. Third, movements in different planes and different positions were measured in this study. Moreover, proprioception, craniovertebral angle, strength and endurance of short cervical flexors and extensors, and pain were measured. These multiple measurements should have allowed for the assessment of physical impairments in headaches. Fourth, patients with cervicogenic headaches were stratified by the onset of their symptoms as could be linked to a traumatic event or not. This should have allowed comparing the magnitude of physical impairment between these groups.

Limitations of the study

This study has some limitations that should be considered when interpreting the findings. First, the patients in the post-traumatic group were not classified based on the nature of the trauma, the site of the trauma, and the extent of post-traumatic impairments. Second, although the findings on the deficits in the active cervical range of motion are informative, the passive range of motion was not investigated in this study. Assessing the cervical passive range of motion could have provided interesting findings [21]. Second, the technique proposed by Loudon et al. was used to measure cervical proprioception in this study [13]. This technique can be less sensitive in detecting deficits in cervical proprioception compared to other techniques such as the one proposed by Revel et al. [23]. Third, the pain scores were self-reported. These scores could be subject to recall and desirability bias.

## Conclusions

Patients with post-traumatic cervicogenic headaches have significant deficits in the cervical active range of motion in the different planes, endurance, and strength of neck flexors and extensors compared to patients with migraine and non-traumatic cervicogenic headaches. Similarly, patients with post-traumatic cervicogenic headaches reported higher affective pain compared to patients with migraine and non-traumatic cervicogenic headaches. The findings of this study indicated that patients with post-traumatic cervicogenic headaches have significantly higher physical impairments compared to patients with non-traumatic cervicogenic headaches. These differences warrant caution when combining data from patients with non-traumatic and post-traumatic cervicogenic headaches.

## References

[1] Jull G (2023). Cervicogenic headache. Musculoskelet Sci Pract.

[2] (2018). Headache Classification Committee of the International Headache Society (IHS) The International Classification of Headache Disorders, 3rd edition. Cephalalgia.

[3] Demont A, Lafrance S, Benaissa L, Mawet J (2022). Cervicogenic headache, an easy diagnosis? A systematic review and meta-analysis of diagnostic studies. Musculoskelet Sci Pract.

[4] Shahwan BS, D'emeh WM, Yacoub MI (2022). Evaluation of computer workstations ergonomics and its relationship with reported musculoskeletal and visual symptoms among university employees in Jordan. Int J Occup Med Environ Health.

[5] (2023). National statistics. https://www.nhtsa.gov/data.

[6] Dumas JP, Arsenault AB, Boudreau G, Magnoux E, Lepage Y, Bellavance A, Loisel P (2001). Physical impairments in cervicogenic headache: traumatic vs. nontraumatic onset. Cephalalgia.

[7] Núñez-Cabaleiro P, Leirós-Rodríguez R (2022). Effectiveness of manual therapy in the treatment of cervicogenic headache: a systematic review. Headache.

[8] Heikkilä HV, Wenngren BI (1998). Cervicocephalic kinesthetic sensibility, active range of cervical motion, and oculomotor function in patients with whiplash injury. Arch Phys Med Rehabil.

[9] Bansevicius D, Pareja JA (1998). The "skin roll" test: a diagnostic test for cervicogenic headache?. Funct Neurol.

[10] Sjaastad O, Fredriksen TA, Pfaffenrath V (1998). Cervicogenic headache: diagnostic criteria. The Cervicogenic Headache International Study Group. Headache.

[11] Rheault W, Albright B, Beyers C, Franta M, Johnson A, Skowronek M, Dougherty J (1992). Intertester reliability of the cervical range of motion device. J Orthop Sports Phys Ther.

[12] Zwart JA (1997). Neck mobility in different headache disorders. Headache.

[13] Loudon JK, Ruhl M, Field E (1997). Ability to reproduce head position after whiplash injury. Spine (Phila Pa 1976).

[14] Braun BL, Amundson LR (1989). Quantitative assessment of head and shoulder posture. Arch Phys Med Rehabil.

[15] Solow B, Tallgren A (1971). Natural head position in standing subjects. Acta Odontol Scand.

[16] Soomro RR, Karimi H, Gillani SA (2022). Reliability of hand-held dynamometer in measuring gluteus medius isometric muscle strength in healthy population. Pak J Med Sci.

[17] Silverman JL, Rodriquez AA, Agre JC (1991). Quantitative cervical flexor strength in healthy subjects and in subjects with mechanical neck pain. Arch Phys Med Rehabil.

[18] Grimmer K (1994). Measuring the endurance capacity of the cervical short flexor muscle group. Aust J Physiother.

[19] Melzack R (1987). The short-form McGill Pain Questionnaire. Pain.

[20] Hawker GA, Mian S, Kendzerska T, French M (2011). Measures of adult pain: Visual Analog Scale for Pain (VAS Pain), Numeric Rating Scale for Pain (NRS Pain), McGill Pain Questionnaire (MPQ), Short-Form McGill Pain Questionnaire (SF-MPQ), Chronic Pain Grade Scale (CPGS), Short Form-36 Bodily Pain Scale (SF-36 BPS), and Measure of Intermittent and Constant Osteoarthritis Pain (ICOAP). Arthritis Care Res (Hoboken).

[21] Bravo Petersen SM, Vardaxis VG (2015). The flexion-rotation test performed actively and passively: a comparison of range of motion in patients with cervicogenic headache. J Man Manip Ther.

[22] Youdas JW, Garrett TR, Suman VJ, Bogard CL, Hallman HO, Carey JR (1992). Normal range of motion of the cervical spine: an initial goniometric study. Phys Ther.

[23] Revel M, Andre-Deshays C, Minguet M (1991). Cervicocephalic kinesthetic sensibility in patients with cervical pain. Arch Phys Med Rehabil.

[24] Watson DH, Trott PH (1993). Cervical headache: an investigation of natural head posture and upper cervical flexor muscle performance. Cephalalgia.

[25] Barton PM, Hayes KC (1996). Neck flexor muscle strength, efficiency, and relaxation times in normal subjects and subjects with unilateral neck pain and headache. Arch Phys Med Rehabil.

